# Spectral Reflectance Can Differentiate Tracheal and Esophageal Tissue in the Presence of Bodily Fluids and Soot

**DOI:** 10.3390/s20216138

**Published:** 2020-10-28

**Authors:** David Berard, Chirantan Sen, Corinne D. Nawn, August N. Blackburn, Kathy L. Ryan, Megan B. Blackburn

**Affiliations:** 1 Tactical Combat Casualty Care Research Department, US Army Institute of Surgical Research, JBSA Fort Sam Houston, TX 78234, USA; berard64@gmail.com (D.B.); cs3180@msstate.edu (C.S.); nawn.cori@gmail.com (C.D.N.); kathy.l.ryan.civ@mail.mil (K.L.R.); 2 Department of Mechanical Engineering, University of Texas at San Antonio, San Antonio, TX 78249, USA; 3 Department of Electrical and Computer Engineering, Mississippi State University, Mississippi State, MS 39762, USA; 4 Blackburn Statistics, LLC, San Antonio, TX 78260, USA; august.blackburn.phd@blackburnstatistics.com

**Keywords:** spectral reflectance, airway management, endotracheal tube placement, tissue detection, tracheal tissue, esophageal tissue, intubation, endotracheal tube misplacement

## Abstract

Endotracheal intubation is a common life-saving procedure implemented in emergency care to ensure patient oxygenation, but it is difficult and often performed in suboptimal conditions leading to high rates of patient complications. Undetected misplacement in the esophagus is a preventable complication that can lead to fatalities in 5–10% of patients who undergo emergency intubation. End-tidal carbon dioxide monitoring and other proper placement detection methods are useful, yet the problem of misplacement persists. Our previous work demonstrated the utility of spectral reflectance sensors for differentiating esophageal and tracheal tissues, which can be used to confirm proper endotracheal tube placement. In this study, we examine the effectiveness of spectral characterization in the presence of saline, blood, “vomit”, and soot in the trachea. Our results show that spectral properties of the trachea that differentiate it from the esophagus persist in the presence of these substances. This work further confirms the potential usefulness of this novel detection technology in field applications.

## 1. Introduction

A fundamental step in front-line emergency medical care is ensuring patient oxygenation. The most common procedure to secure a patient’s airway is endotracheal intubation (ETI), in which an endotracheal tube (ETT) is inserted and secured in the trachea. Caregivers face a variety of challenges when attempting ETI, including the difficulty of the procedure itself, environmental variables, patient condition and presentation, as well as obstructive anatomical features of the patient. These challenges increase the rate of failed intubations resulting in patient complications up to and including death [1,2]. ETT placement generally begins when the caregiver visualizes the upper airway using a laryngoscope. However, patient anatomy, injuries such as maxillofacial trauma, swelling, hemorrhage, and other environmental contaminants can increase the difficulty of visualization in as much as 50% of cases in the prehospital setting [3,4]. Due to these difficulties, an array of complications may arise including, hypoxia, hypotension, as well as cardiac arrest resulting from failed intubations [2,5].

Esophageal intubation is one type of failure in ETI in which the ETT is placed in the esophagus rather than the trachea. Habib et al. found this to occur in up to 25% of patients arriving at the emergency department, and if undetected, is fatal [6]. As a precaution against undetected misplacement, the American Heart Association recommends use of a confirmatory procedure following any ETI [7]. Current methods for confirmation include direct visualization of proper ETT placement, which may not be possible due to aforementioned challenges, and the clinically preferred method of end-tidal carbon dioxide monitoring (ETCO2). Although a recent survey revealed that 95% of respondents in the New York State area had capnography and CPR devices aboard ambulances [8], these resources may still not be available in far-forward areas, such as a military setting, and even when available have been found to be underutilized [9]. Additionally, confirmatory methods may require an interruption of patient care or extra equipment that may not be available especially when only a single caregiver is present. Given the time sensitive nature of emergency care, the potential delays and distractions of performing these confirmations can be detrimental to patient outcomes.

Therefore, there is a proven need for an easy, fast, and nonvisual detection mechanism to confirm tracheal placement. We have previously shown that tracheal and esophageal tissues reflect white light differently and produce significantly different spectral profiles in ex vivo and in vivo settings [10,11], as well as in human cadavers [10]. Specifically, tracheal tissue contains a local maximum reflectance intensity at 561 nm with two local minimum intensities at 543 and 578 nm that are not present in esophageal tissues. Thus, the ratios of the reflectance intensities at these key wavelengths, which we refer to as Ratio B (561/543) and Ratio Y (561/578), can differentiate between healthy esophageal and tracheal tissues. We have also demonstrated that these spectral signatures continue to differentiate between esophageal and tracheal tissues in vivo under hypoxic conditions [12]. However, contaminants in the trachea are common in the event of traumatic injury, therefore, it is important to determine if the spectral signatures present in the trachea persist in the presence of contaminants. We hypothesized that spectral reflectance would still differentiate tracheal and esophageal tissues when saline, blood, a simulant of vomit, and soot are present in the trachea.

## 2. Materials and Methods

### 2.1. Sample Collection and Spectra Collection

Research was conducted in compliance with the Animal Welfare Act, the implementing Animal Welfare regulations, and the principles of the Guide for the Care and Use of Laboratory Animals, National Research Council. The U.S. Army Institute of Surgical Research Institutional Animal Care and Use Committee approved all research conducted in this study; assigned identification code A-18-012 and approved on 18 January 2018. The facility where this research was conducted is fully accredited by the AAALAC.

**Figure 1:**
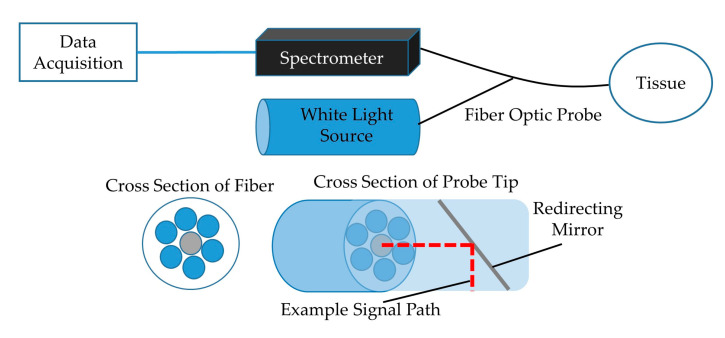
Block diagram of the experimental setup showing data acquisition module and light source connected to fiber optic probe. Cross-section of fiber with emitting and receiving fibers and tip showing mirror with 90° signal redirection included (adapted from previous publication [11]).

The trachea and esophagus were excised from 16 euthanized swine to be studied ex vivo. Once removed, the tissues were stored in a freezer until use and permitted to defrost in a refrigerator for not less than 12 h prior to testing. To collect spectral reflectance data, a custom-designed, 200 µm core, fiber optic probe from Gulf Fiberoptics (R200-7-UV-VIS) was placed into each sample trachea and esophagus (Figure 1). The fiber optic probe contained a centrally located illuminating fiber that carried non-ionizing light emitted from an 8.8 mW LLS-COOL-WHITE halogen light source by Ocean Optics down the 2 m long probe cable where it was redirected 90° by a mirror located at the distal tip of the probe and shown on the adjacent tissue. This design allowed scans to be done with the probe aligned parallel to the tissue and eliminated the need to dissect the tissue structure in order to orient the probe normal to the tissue surface. The probe also contained six signal receiving fibers surrounding the emitting fiber, as shown in Figure 1, that transported the reflected light from the tissue back to a Photon Control SPM-002-DT spectrometer sensitized for 420–1070 nm wave lengths with 2048 × 14 pixel Hamamatsu back-thinned CCD (S9840), and spectral properties were collected from the tracheal and esophageal lumens with the Ocean View Specsoft software package. A “dark” calibration scan was captured after placing the probe in a near perfectly dark environment, and a “light” scan was captured by turning on the light source and placing the probe in a white Ocean Optics FIOS-1 calibration box. Each capture was collected by averaging five subsequent exposures of 5 ms each. Spectral properties of each trachea were measured without contaminants and in the presence of various substances and compared to clean esophagus tissue.

### 2.2. Experimental Group 1

The first set of experiments, *n* = 10, included a 0.9% saline solution, blood (collected during experimental hemorrhage), and simulated “vomit” (cream of mushroom soup), measured in that order. For each swine, three spectral captures were recorded for each contaminant (baseline, saline, blood, and “vomit”) in the trachea followed by three spectral captures of the non-contaminated esophagus, resulting in 24 spectral captures per swine in total. Previous work demonstrated that the spectral characteristics of interest were independent of the location within a particular tissue, so each capture was collected at random locations within the treated area [11]. Each substance was introduced to the trachea via syringe and the tissue was thoroughly rinsed and dried between each test. Any sign of tissue injury created during handling, including abrasion, constituted exclusion of that sample. Saline was not captured for one swine and one measurement of “vomit” in trachea was missed in each of two swine. There were a total of 220 spectral captures for this group of swine.

### 2.3. Experimental Group 2

**Figure 2:**
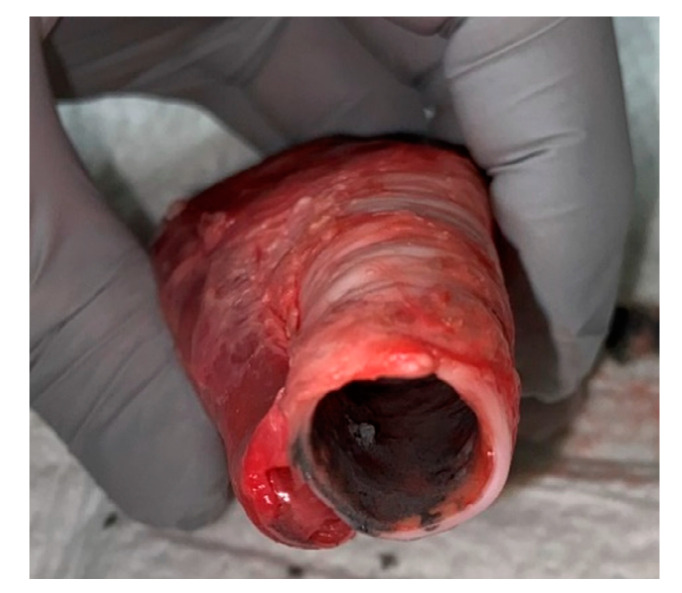
Trachea sample with soot applied prior to spectral collection.

A second group, *n* = 6, was used to investigate spectral reflectance in the presence of soot in the trachea. Soot was collected by capturing burned paper and reconstituting in saline to make a paste. Figure 2 shows an example of a trachea sample with soot applied. The procedure described above was followed for the application of soot. Twelve spectral captures per swine were taken for a total of 72; to include three baseline measurements in the trachea and esophagus followed by three trachea in the presence of soot and three clean esophagus.

### 2.4. Spectral Reflectance Processing

The reflectance, absorbance, and amplitude spectra were exported from the Specsoft software as text files and imported into MATLAB for post-analysis. Each capture was identified numerically, according to the experiment, and classified as tracheal or esophageal based on the relevant tissue type. All tracheal and esophageal spectra were first plotted separately to inspect for signal fidelity. Previous work identified 500–650 nm as the range of interest [13], and therefore all spectra were cropped to focus on that region prior to signal processing. Based on previous studies identifying 543, 561, and 578 nm as the wavelengths most useful in distinguishing tracheal and esophageal tissues [13,14], the amplitude values at these wavelengths were extracted for each capture and plotted to visualize the distribution.

### 2.5. Statistical Analysis

Spectral reflectance was normalized across captures by dividing each spectral reflectance datapoint by the average spectral reflectance across the range of measurements from 500 to 650 nm. This procedure produced an average normalized spectral reflectance of 1 for each capture and retains relative differences between reflectance at different wavelengths. For each capture, the spectral reflectance datapoints were smoothed using kernel regression. Specifically, we used a Gaussian kernel with a bandwidth chosen using the method of Racine and Li [15], implemented within the np package in R [16]. Smoothed reflectance for the wavelengths 543, 561, and 578 nm were extracted for further analysis. Ratio B (561/543) and Ratio Y (561/578) were calculated and used as the primary measures of interest.

The spectral reflectance ratios of interest, Ratio B and Ratio Y, were modelled independently using linear mixed models. The spectral reflectance ratio was treated as the dependent variable. Individuals (swine) were treated as random effects in order to account for non-independence due to multiple measurements per individual. Contaminants (baseline, saline, blood, “vomit”, and soot) were treated as random effects and the location of the probe (esophagus vs. trachea) was treated as a fixed effect. Receiver operator characteristic (ROC) curves were used to characterize the diagnostic ability of the spectral reflectance ratios to distinguish esophagus from trachea. Differences in the area under the curve (AUC) between ROC curves were tested using the method described by DeLong et al. [17].

## 3. Results

**Figure 3:**
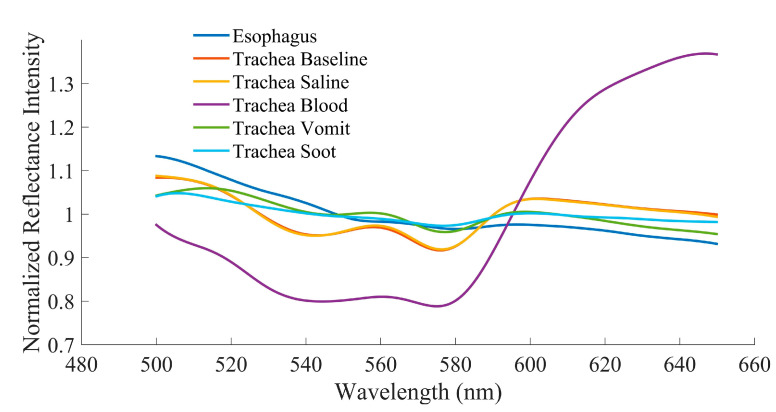
Averaged reflectance spectra at baseline and in the presence of various body fluids for esophageal and tracheal tissues.

Spectral scans of 96 distinct points across 16 clean tracheal and esophageal samples were collected to establish a baseline measurement. At baseline, the characteristic peak at 561 nm along with the adjacent troughs at 543 and 578 nm were present in the trachea while no such features were present in the esophagus, as expected (Figure 3). Tracheal scans taken in the presence of saline, blood, “vomit”, and soot continued to show the presence of a spectral peak at 561 nm and accompanying troughs at 543 and 578 nm (Figure 3).

**Figure 4:**
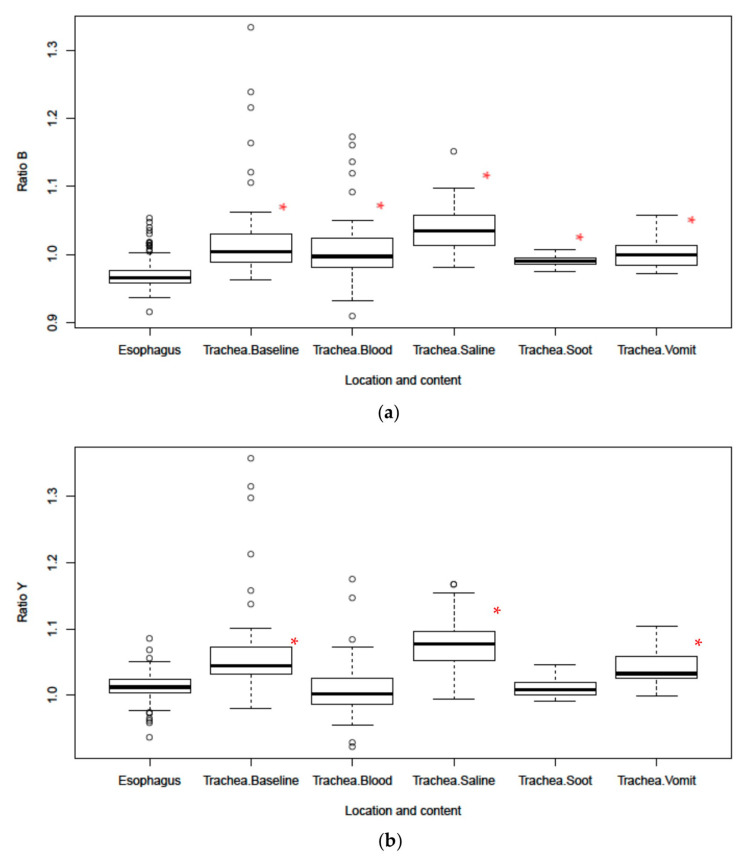
(**a**) Boxplot of Ratio B and (**b**) Ratio Y for tracheal and esophageal tissues in the presence of body fluids and soot. Dots in the boxplots are outliers, defined as being 1.5 times the interquartile range beyond the first or third quartiles. * Represents statistical significance vs. esophagus.

**Figure 5:**
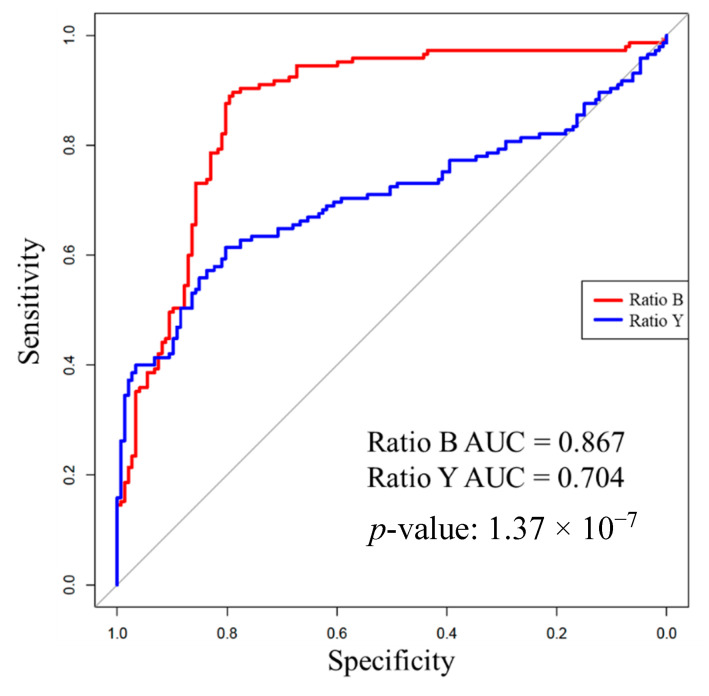
Receiver operator characteristic curves including all Ratio B and Ratio Y measurements in this study with corresponding area under the curve (AUC) values. The AUC for Ratio B is statistically significantly greater than for Ratio Y. In this context, specificity and sensitivity are reported according to correct identification of trachea.

The baseline scans affirmed the results from previous work that identified Ratio B (561/543) and Ratio Y (561/578) as reliable markers to differentiate between tracheal and esophageal tissues (Figure 4). Tracheal tissue had significantly larger mean Ratio B values for every substance tested when compared to esophageal tissue. The Ratio Y mean was also significantly larger in tracheal than esophageal tissue at baseline and in the presence of saline and “vomit”. However, the Ratio Y mean was only nominally larger with soot in the trachea and was smaller with blood in the trachea. These results are reflected in the area under the receiver operator characteristic curves for each ratio, as shown in Table 1 and Figure 5. Overall, Ratio B has a better sensitivity and more specificity in all settings as indicated by the area under the receiver operator characteristic curves (Table 1). This indicates an inability of Ratio Y to distinguish between the two tissues if blood or soot is present.

## 4. Discussion

We evaluated the capabilities of a novel biosensor for detecting proper ETT placement during ETI. Using a fiber optic probe, the spectral reflectance characteristics of tracheal and esophageal tissues can be analyzed and used to differentiate between them, even in the presence of bodily fluids in the trachea which may be encountered in patients with traumatic injury.

Incorporation of a spectral reflectance sensor into the ETT would give providers continuous feedback while performing ETI and provide continuous monitoring to alert providers should the ETT become dislodged during transport. This novel approach shows promise of being more reliable than the current standard of care, ETCO2 monitoring, particularly in pre-hospital settings. ETCO2 monitoring is possible through different techniques including colorimetry, capnography, or capnometry and effectively reduces the amount of ETT misplacements that go undetected [18,19]. The colorimetric approach uses a single-use device housing a chemically treated paper sample. The paper sample reacts when exposed to CO_2_ resulting in a color change that can be observed by the caregiver. Unfortunately, the colorimetric method can be slow and subject to user interpretation. Mouth-to-mouth resuscitation, contamination by acidic substances, and various medications can also cause false positive test results [7,20]. The colorimetric sensor is also not capable of providing continuous monitoring, which is problematic as ETTs may become dislodged after initial proper placement [9]. Capnography and capnometry are more quantitative methods that measure CO_2_ concentration in exhaled breath [21]. However, the equipment required for these continuous monitoring approaches may be unavailable or underutilized in a prehospital setting. Capnometry in particular relies on higher CO_2_ concentrations which may be hindered in instances of cardiac arrest with reduced blood flow and low CO_2_ levels [22,23]. If placed into the ETT itself, spectral reflectance sensors may offer better resilience to the aforementioned conditions and provide continuous placement monitoring in places where ETCO2 monitoring is not feasible or unavailable.

Our previous studies identified wavelength ratios B (561/543) and Y (561/578) which can be used to capture the relative prominence of the tracheal peak [10]. However, to date, the ability of these ratios to distinguish between tissue types had not been investigated in the presence of bodily fluids in the trachea likely to be encountered during ETI during traumatic injury, including burn inhalation injury where hot gases and soot particles may be introduced into the airway. Baseline results confirmed previous findings that both ratios were significantly different between the two tissues in an ex vivo swine model [10,11,13]. With saline in the trachea, both ratios remain capable of discriminating between tissue types. This was expected since liquid water is almost perfectly transparent and thus would negligibly affect the reflectance intensities of the tissue. Blood had the greatest impact on the spectra signature characteristic of the trachea, especially with regard to Ratio Y. Figure 3 shows that there is high absorption of wavelengths <600 nm in the presence of blood. Though a local peak and the adjacent troughs are still visually identifiable, they are dramatically blunted in comparison to the tracheal spectra with other substances. This can most likely be attributed to the fact that oxyhemoglobin has relatively high absorption in this wavelength range and low absorption in the 630–700 nm range, which results in its red color [24,25]. Possible higher concentrations of oxyhemoglobin in the tissue layers closer to the surface may also account for the characteristic differences between tracheal and esophageal tissue curves overall [10]. Despite this, Ratio B still differentiated between tracheal and esophageal tissue in the presence of blood, suggesting that its use in a sensor system could overcome this greater absorption. Interestingly, even though tracheal reflectance was impacted by blood, the spectral sensor was capable of differentiating the tissue types in the presence of “vomit” and soot. The relatively opaque substance used for “vomit” and dark color of soot had very little effect on the tracheal reflectance profile.

Overall, Ratio B demonstrated a more robust reliability for distinguishing between tracheal and esophageal tissues than Ratio Y as evidenced by the significantly higher ratios and AUC values for all substances. The overall ROC curves show that Ratio B is significantly closer to the perfect classification, 100% sensitivity (no false negatives) and 100% specificity (no false positive), as compared to Ratio Y. Further studies are warranted to determine whether Ratio B alone would be sufficient or if slightly altered wavelength values might provide greater acuity than the currently selected values for Ratio Y, especially in the presence of blood. Additional research is also needed to determine an optimal clinical strategy for using this information. In the absence of perfect discrimination, which would be reflected by an area under the receiver operator characteristic curve of 1, there are different clinical strategies that should be considered. For example, a threshold for Ratio B could be set to be highly specific or highly sensitive for tracheal placement or to strike a balance between sensitivity and specificity. While we focused on Ratios B and Y in this work, it is possible that other analytical approaches for classifying spectra produced by this device as representing placement in the trachea or esophagus may be superior.

Only four substances were examined in this study with only one substance present at a time. It is also possible that sequentially measuring different contaminants in the same tissue could alter the measurements somehow. We consider this possibility unlikely given the cleaning procedure between contaminants and that the characteristic spectral feature of the trachea persists. Future work may need to include mixtures of these substances as well as others. These four are some of the most likely, but the authors recognize the potential for a multitude of contaminants and their potential effect on spectral characteristics of tissues. Additional studies can also be done on tissue that has undergone trauma since it is currently unknown how certain injuries may impact the reflectance spectra. Inhalation injuries are particularly common in instances of fire emergencies and explosions and often require intubation. It is possible that these effects of inhalation injury on the spectral profile may be quantified as a diagnostic tool for quantifying the severity of the injury. Future studies are also necessary to verify that these spectral properties hold true in living humans. The cost of a clinically used spectral device has not yet been evaluated, however, the available technology can be priced competitively when compared to current small form factor ETCO2 devices. The small footprint and light weight of the components makes this preferable to other larger equipment when these metrics are paramount.

## 5. Conclusions

We showed that certain ratios of reflectance values at specific wavelengths can be used to distinguish between tracheal and esophageal tissues in an array of conditions likely to be encountered in emergency trauma scenarios. Although the spectral profile of tracheal tissue was altered slightly by the different substances tested, the characteristics distinguishing it from the esophagus remained quantitatively detectable. Ratio B (561/543 nm) proved to be a robust metric that can discriminate tracheal tissue in the presence of saline, blood, “vomit”, and soot with reliable AUC values of ROC curves and statistically significant differences in mean ratio values. The absorption properties of blood are most likely responsible for the low reflectance values for wavelengths below 600 nm. In spite of this, some of the qualitative characteristics are still visible, and Ratio B remains significantly different. However, the combination of the general negative slope of esophageal tissue within the area of interest and the reduced intensity of the trachea in the presence of blood and soot caused Ratio Y to no longer reliably differentiate the tissues. Taken together, these results strengthen the potential of spectral reflectance to be used for confirming proper ETT placement in prehospital settings. The probe and accompanying accessories can be integrated into already existing airway devices such as a bougie or ETT.

## Figures and Tables

**Table 1:** Area under the receiver operator characteristic curves for differentiating trachea from esophagus using Ratio B and Ratio Y.

Substance	Ratio	
	B	Y
Overall	0.867	0.704
Baseline	0.891	0.871
Saline	0.947	0.878
Blood	0.763	0.364
“Vomit”	0.873	0.801
Soot	0.847	0.557

## Animal Statement

Research was conducted in compliance with the Animal Welfare Act, the implementing Animal Welfare regulations, and the principles of the Guide for the Care and Use of Laboratory Animals, National Research Council. The U.S. Army Institute of Surgical Research Institutional Animal Care and Use Committee approved all research conducted in this study; assigned identification code A-18-012 and approved on 18 January 2018. The facility where this research was conducted is fully accredited by the AAALAC.
